# A girl with primary pigmented adrenocortical disease (PPNAD): challenges in diagnosis and management

**DOI:** 10.1186/1687-9856-2013-S1-P130

**Published:** 2013-10-03

**Authors:** Karn Wejaphikul, Vichit Supornsilchai, Suttipong Washarasindhu, Taninee Sahakitrungruang

**Affiliations:** 1 Endocrine Unit, Department of Pediatrics, Faculty of Medicine, Chulalongkorn University, Bangkok, Thailand 10330

## Introduction

PPNAD is a rare cause of pediatric Cushing syndrome (CS). Diagnosis and management of this condition remains challenging. Although, there are diagnostic approaches and various tests to identify the cause of CS, but these tests appears to have incomplete diagnostic accuracy.

## Clinical case

A 2-year-old, previously healthy girl presented with rapid weight gain for 5 months. She had Cushingoid appearance, acne, hirsutism, hypertension, and normal pre-pubertal female genitalia. Her weight was 16.9 kg (>97^th^ percentile) and height was 85 cm (3^rd^ percentile). Serum cortisol levels revealed loss of diurnal variation (35.0 and 30.1 μg/dl at 8.00 am and 11.00 pm, respectively). ACTH levels were unsuppressible at 22.4 (1^st^) and 20.5 pg/ml (2^nd^). Testosterone and DHEAS levels were undetectable. Abdominal USG were done twice. The first study showed enlargement of bilateral adrenals (right 1.6x1.2 cm and left 1.7x1.4 cm), but the subsequent one was unremarkable. Pituitary MRI was normal. CT chest included neck and abdomen revealed normal adrenal glands without mass. High dose dexamethasone suppression test (HDDST) revealed unsuppressible cortisol levels (baseline 51.9 and post-HDSST 47.2 μg/dl). Repeated ACTH levels were 13.6 and 15.1 pg/ml, suggested ACTH-independent CS. CT abdomen was reviewed by an adrenal radiologist expert and a small nodule (3-4 mm in size) of the right gland was identified, thus micronodular adrenocortical hyperplasia (MAD) was suspected. Laparoscopic right adrenalectomy was done. The right adrenal gland had normal gross findings, but histopathology revealed nodular thickening of the cortical tissue and internodular atrophy, which were compatible with PPNAD. After surgery, the features of CS were gradually subsided and cortisol levels were steadily declined to normal.

## Clinical lessons

Adrenal CS is more common in this age group, but initial hormonal profiles and imaging studies misled us. Non-suppressed ACTH levels could be found in adrenal CS. Other tests (CRH/HDDST) are useful to guide the diagnosis. Imaging studies should be best interpreted by experienced radiologist. Normal adrenal glands are not uncommon in MAD and its variant, PPNAD. Nodular thickening and internodular atrophy are pathognomonic of PPNAD even if no pigmentation seen in gross findings. PPNAD is caused by *PRKARIA* gene mutation which may present as isolated form or is associated with Carney’s complex. Genetic testing is important because this disorder is bilateral involvement. Thus, unilateral adrenalectomy may be inadequate. Carefully follow-up is needed and contralateral adrenalectomy may be required in the future.

## Conclusion

Diagnosis and management of CS are challenges in pediatric endocrine practice, and should be best done by a team experienced in managing pediatric CS. Accurate diagnosis and localization is the key to success in managing these cases.

